# Quantifying Hand Motion Complexity in Simulated Sailing Using Inertial Sensors

**DOI:** 10.3390/s24206728

**Published:** 2024-10-19

**Authors:** Gurdeep Sarai, Prem Prakash Jayaraman, Nilmini Wickramasinghe, Oren Tirosh

**Affiliations:** 1 School of Health Sciences, Swinburne University of Technology, Hawthorn, VIC 3122, Australia; 2 School of Science, Computing and Engineering Technologies, Swinburne University of Technology, Hawthorn, VIC 3122, Australia; 3 School of Computing, Engineering and Mathematical Sciences, La Trobe University, Bundoora, VIC 3086, Australia; 4 School of Health and Biomedical Sciences, Royal Melbourne Institute of Technology, Bundoora, VIC 3082, Australia

**Keywords:** approximate entropy (ApEn), inertial measurement units (IMUs), sailing simulation, motion time-series analysis, handedness

## Abstract

The control of hand movement during sailing is important for performance. To quantify the amount of regularity and the unpredictability of hand fluctuations during the task, the mathematical algorithm Approximate Entropy (ApEn) of the hand acceleration can be used. Approximate Entropy is a mathematical algorithm that depends on the combination of two input parameters including (1) the length of the sequences to be compared (m), and (2) the tolerance threshold for accepting similar patterns between two segments (r). The aim of this study is to identify the proper combinations of ‘m’ and ‘r’ parameter values for ApEn measurement in the hand movement acceleration data during sailing. Inertial Measurement Units (IMUs) recorded acceleration data for both the mainsail (non-dominant) and tiller (dominant) hands across the X-, Y-, and Z-axes, as well as vector magnitude. ApEn values were computed for 24 parameter combinations, with ‘m’ ranging from 2 to 5 and ‘r’ from 0.10 to 0.50. The analysis revealed significant differences in acceleration ApEn regularity between the two hands, particularly along the Z-axis, where the mainsail hand exhibited higher entropy values (*p* = 0.000673), indicating greater acceleration complexity and unpredictability. In contrast, the tiller hand displayed more stable and predictable acceleration patterns, with lower ApEn values. ANOVA results confirmed that parameter ‘m’ had a significant effect on acceleration complexity for both hands, highlighting differing motor control demands between the mainsail and tiller hands. These findings demonstrate the utility of IMU sensors and ApEn in detecting nuanced variations in acceleration dynamics during sailing tasks. This research contributes to the understanding of hand-specific acceleration patterns in sailing and provides a foundation for further studies on adaptive sailing techniques and motor control strategies for both novice and expert sailors.

## 1. Introduction

The subtle dance of our fingers, the precise movements of our hands, fine motor control is woven into nearly every aspect of human life. It involves the coordination of small muscles in the eyes, hands, and fingers, leading to dynamic movements such as reaching and grasping [1]. This intricate coordination extends beyond everyday tasks, playing a crucial role in various physical activities. In the context of sailing, achieving optimal wind utilization necessitates fine motor control for precise adjustments to the sails [2]. These adjustments can be interpreted as “reaching” and “side-to-side” tasks, which are critical for the performance and safety of sailors. Investigating these movements and handedness provides valuable insights into both healthy individuals and those with various medical conditions [3].

Handedness refers to the tendency to use one hand over the other and is linked to brain lateralization, with each hemisphere controlling movement in the opposite hand [4]. It includes right-handedness, left-handedness, and ambidexterity. Handedness consists of two components: hand preference (using the preferred hand for tasks) and hand performance (differences in abilities between hands) [5,6]. Most individuals develop a hand preference from a young age, influenced by genetic and environmental factors [7]. Generally, the dominant hand is more adept at performing skilled tasks that require precision and fine motor control, such as steering or aiming [8], whereas the non-dominant hand is frequently tasked with supporting roles that involve broader, less refined motions, such as holding or stabilizing. However, unskilled tasks like reaching may show no significant difference between both hands [9,10]. This distinction between hand functions can be impactful when assigning roles in complex activities like sailing, where the precision and sensitivity required for steering (tiller control) are better suited to the dominant hand, while the less intricate but forceful maneuvers needed to handle the mainsail are effectively managed by the non-dominant hand. However, the relationship between hand skill and preference remains complex and not fully understood, potentially due to variations dependent on experience and environmental demands.

Research on motor skill lateralization has focused on how specialization contributes to differences in hand preference and performance. Hemispheric asymmetry, the division of control between brain hemispheres, has also been explored. For instance, Nelson et al. [11] investigated handedness and limb control in adults, finding mixed support for the dynamic dominance hypothesis, with right-handers generally performing better. Mutha et al.’s [12] study challenges conventional handedness views, suggesting specialized mechanisms in each arm–hemisphere system, with the dominant hemisphere aiding predictive control and the non-dominant one stabilizing the arm at a goal position. Boulinguez-Ambroise et al. [13] highlighted that although hemispheric dominance is an important factor, it does not fully explain the variations observed in performance. This suggests that additional cognitive and motor elements also contribute to the underlying mechanisms. Overall, the interplay between hemispheric asymmetry and motor skill specialization is complex and multidimensional, necessitating further research to comprehensively understand its implications for hand preference and performance.

Hand function assessments frequently rely on questionnaires, which can introduce subjectivity and bias. As noted by Mcsp and Dipcot [14], and further supported by Peters [15], self-reported measures often fail to accurately capture an individual’s actual hand capabilities due to personal perceptions and emotional states [14,15]. This subjectivity can lead to inconsistent results, particularly in clinical settings where objective measures are crucial. Bear-Lehman and Abreu [16] also highlight that questionnaires may overlook essential aspects of hand function, such as dexterity and strength [16]. This highlights the importance of quantitative data for studying movement patterns between hands as well as choosing the appropriate technology and measures for accurately capturing fine motor control and dynamic hand movements [17].

Entropy, a measure of system randomness or uncertainty, is one such measure [18]. Numerous studies have used Inertial Measuring Units (IMUs) to measure reaching, applying kinematics and kinetics techniques. However, analyzing three-axis motion time-series data for hand dominance differences in sailing has been overlooked, likely due to the complexity of such data.

This study seeks to address the gap in research by examining the control of the hand movement during multi-directional sailing simulation. Movement control of the hand can be quantified using the ‘regularity statistic’ Approximate Entropy (ApEn) that quantifies the unpredictability of fluctuations in hand acceleration time series. ApEn reflects the likelihood that ‘similar’ patterns of observations will not be followed by additional ‘similar’ observations. Acceleration time series containing many repetitive patterns have a relatively small ApEn; a less predictable and more complex process has a higher ApEn value. The algorithm for computing ApEn depends on the ‘m’ and ‘r’ input parameters. The parameter ‘m’ specifies the pattern length, and ‘r’ defines the criterion of similarities. The aim of this study is to identify the proper combinations of ‘m’ and ‘r’ parameter values for ApEn measurement in the hand movement acceleration data during sailing. The findings will provide valuable insights to inform future investigations on the appropriate selection of parameters for ApEn analyses in sailing.

## 2. Method

### 2.1. Participants

The study involved 10 participants, aged 18 to 30 years, who volunteered after an initial screening process. Eligibility criteria included no prior experience in sailing, and the absence of current or chronic health conditions and physical disabilities or injuries. Individuals with clinically significant diseases or disorders were excluded. Participants underwent further screening using the Adult Pre-Exercise Screening System [19] tool to identify those susceptible to adverse exercise-related events and those showing signs of known illnesses. Hand dominance was determined using the Edinburgh Handedness Inventory [20], revealing that eight participants were right-handed and two were left-handed. Prior to the experiment, all participants received an information sheet detailing the research process and provided informed consent. Ethical approval was granted by the Swinburne University Human Research Ethics Committee (SUHREC—Ref: 20226403-12100).

### 2.2. Equipment

**Figure 1:**
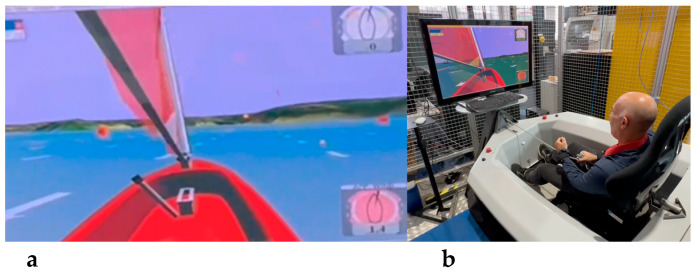
(**a**) The virtual HUD display in the sailing simulator, also showing the triangular course. (**b**) A participant during a trial with the sailing simulator.

The study utilized a VSail-Trainer^®^, designed by the company Virtual Sailing Pty Ltd. (Virtual Sailing, Parkville, VIC, 3052, Australia). It comprises one boat hull (size length: 230 cm, breadth: 150 cm) based on the Hansa 303 model boat (see Figure 1). The sailor controls the simulator in a seated setup, controlling the virtual Hansa dinghy’s course via a centrally located joystick and managing speed through the mainsheet. A 42-inch screen displayed the virtual course, while sensors tracked the heel angle, tiller angle, and mainsail tension. This real-time data enabled adjustments to the simulated sailing environment, including wind conditions, gusts, and course variations.

The experimental setup was designed to simulate the ergonomics and control mechanisms of seated dinghy sailing, specifically the Hansa dinghy. A centrally positioned joystick is a common adaptation in accessible sailing for individuals with physical disabilities or those new to sailing. This choice aimed to minimize physical strain and enhance participant engagement.

**Figure 2:**
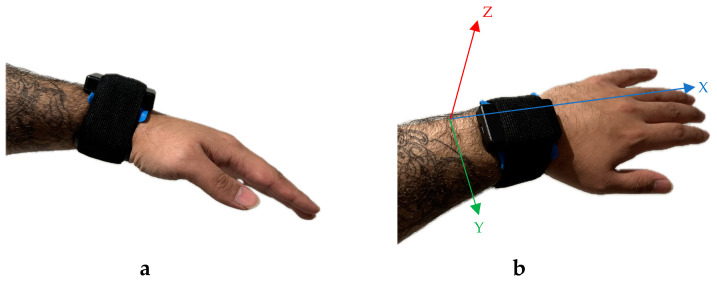
The placement of the IMU sensors on the left and right wrist. IMU reference: the X-axis is pointing forwards, the Y-axis is pointing laterally, and the Z-axis is for the depth. (**a**) Lateral View. (**b**) Point of View.

This study used 2 wireless IMUs, with tri-axial accelerometers (YEI 3-space sensor, Yost Labs, Portsmouth, OH 45662, USA) to capture continuous hand acceleration during sailing. These lightweight sensors (35 mm × 60 mm × 15 mm, 28 g) were secured to participants’ hands using Velcro straps (see Figure 2). Raw acceleration was sampled at 150 Hz (±16 g range) and captured using in-house Python software (version 2.7). The IMU has ±1° orientation accuracy for dynamic conditions, with <0.08° resolution and 0.085° repeatability across all axes.

### 2.3. Multi-Directional Sailing Task

Participants performed the multi-directional sailing simulation using both dominant and non-dominant hands. The course was based on the Sydney 2000 Olympic Games triangle course in Sydney Harbor, designed to challenge participants with upwind and downwind segments. The sailing simulation course commenced with a standardized 30 s start protocol, characterized by northerly winds of 12 knots blowing towards the south. While the simulator dynamically adjusted to user input, ensuring a realistic sailing experience, each participant completed the same pre-defined course involving two tacks and one gybe. To further standardize the sailing task, a “ghost” boat, representing the ideal course trajectory, was incorporated into the visual display. This provided participants with a real-time visual guide for the pre-defined course, ensuring consistent navigational cues throughout the experiment.

This consistency in course maneuvers ensured that all participants encountered identical navigational challenges. Variations in individual performance stemmed from differences in the tiller steering, mainsail trim (specifically twin-tail adjustment), and overall reaction to the simulated conditions, rather than variations in the course itself.

### 2.4. Procedure

Participants were equipped and strapped with an IMU on both wrists to measure accelerations during the sailing task (see Figure 2). Prior to testing, participants had a familiarization session to familiarize themselves with the V-Sail simulator. During the familiarization session, participants received instruction on sailing techniques, including the use of the Heads-Up Display (HUD), which provided real-time data on the wind direction and strength, twin tails’ alignment, boat angle, and course details. The participants were also instructed on the placement of hands, where the dominant hand was typically assigned to the tiller, as steering requires precise, fine motor adjustments. The non-dominant hand was tasked with adjusting the mainsail, a function that, while critical, does not demand the same level of precision as steering in the beginning stages. This division of labor reflects common beginner sailing practices, where the dominant hand’s dexterity is leveraged for steering, and the non-dominant hand supports sail handling, helping the participant during the initial learning process.

During the familiarization session, participants were taught to steer the boat using the tiller, navigate to specific objects, and maneuver around obstacles. They then learned to adjust the mainsail to optimize the sail angle for speed and maneuverability. After mastering basic sailing techniques, participants practiced specific maneuvers such as tacking and gybing, crucial for sailing against the wind or overcoming headwinds. The final stage involved managing the heeling angle using pneumatic rams to reduce water resistance and improve speed and control. The instructional procedures during the familiarization session were standardized across all participants to ensure that the duration of practice and the feedback provided were consistent across participants to eliminate any confounding effects.

Following the familiarization session, participants performed the testing session where hand acceleration data were captured. During the testing session, participants were required to complete 5 laps of the triangular course, navigating outside the buoys, and cross the finishing line as quickly as possible. The various performance measures were collected using the wireless IMUs.

### 2.5. Entropy Measures of Acceleration Time-Series Data

The computation of ApEn, a pivotal metric in assessing motor control complexities, hinges on several intricate parameters crucial for a meaningful analysis [21]. Among these, ‘m’ and ‘r’ are the primary determinants. The parameter ‘m’ signifies the number of sequences being compared for similarity, i.e., m = the length of embedding vectors, while ‘r’ sets the threshold for considering sequences as similar. Additionally, secondary determinants such as ‘N’ (number of data points) ensure a reliable estimate, while the time delay establishes the temporal gap between compared points. The window size influences the analysis scope by determining the portion of data analyzed at any given time. Data normalization is employed to remove scale-induced bias. These parameters collectively ensure the reliability and relevance of entropy calculations, requiring adjustments to align with specific research objectives and the nature of the data.

Our study primarily concentrates on the primary determinants ‘m’ and ‘r’, as these are directly modifiable and crucial for computing ApEn. These factors are essential for balancing the identification of true complexity against the risk of misinterpretation due to noise or inadequate data capture [21]. While other parameters like the N (total number of data points), time delay, window size, and data normalization play supportive roles, they either pertain to the broader study design, pre-processing requirements, or specific considerations of the ApEn algorithm.

In the context of our research using a sailing simulator, identifying and fine-tuning ‘m’ and ‘r’ are paramount before addressing secondary determinants. A well-chosen ‘m’ ensures that the complexity analysis captures relevant hand patterns, while an appropriately set ‘r’ distinguishes true behavioral variability from random noise. This allows for a better understanding of motor control stability or irregularity in individuals during the sailing simulator task.

For reliable entropy results, ‘m’ is typically selected as 2, with occasional use of 3 [22,23,24]. However, we decided to extend this to 5 to explore the potential influence of a higher embedding dimension on entropy measures in our sailing simulator task. These ‘m’ values enable the analysis of sequential complexity from basic (m = 2, three consecutive data points) to advanced (m = 5, patterns of six consecutive data points), ensuring the capture of both simple repetitive patterns and more complex structures in the data.

Typically, values of ‘r’ like 0.1 or 0.2 are common [25,26]. However, due to the unique application of the sailing simulator, we explored higher values up to 0.4. The chosen ‘r’ values (0.1 to 0.4) define the tolerance spectrum for matching data segments. A lower ‘r’ (0.10) enhances sensitivity to fine details, detecting subtle variations, while a higher ‘r’ (0.40) smooths over minor differences, aiding in recognizing broader patterns and reducing sensitivity to noise.

### 2.6. Statistical Procedures

The data processing procedures in this study followed well-established protocols to ensure an accurate analysis of the Inertial Measurement Unit data collected during the sailing simulation tasks.

1. The raw acceleration data from the IMUs were processed to remove noise and baseline drift [27].
2. A zero-phase Butterworth high-pass filter with a cut-off frequency of 0.3 Hz was applied to the acceleration data to eliminate low-frequency noise, ensuring that only the relevant motion data were retained for a further analysis.
3. Following the high-pass filtering, the data underwent smoothing using a third-order Savitzky–Golay filter with a frame size of 41 points. This filter was chosen to preserve the features of the data while reducing high-frequency noise, making it suitable for the entropy analysis.
4. Approximate Entropy (ApEn) values were then calculated, employing the method described by [28]. ApEn was computed using varying combinations of the embedding dimension ‘m’ (values 2 to 5) and the similarity threshold ‘r’ (values 0.1 to 0.5), applied across all three axes (X, Y, Z), and the vector magnitude (Vm) of the hand motion data. The approx_entropy function from the R ‘pracma’ library was used to perform these calculations.
5. A one-way repeated measures ANOVA was conducted to statistically evaluate the significance of differences in Approximate Entropy values between the tiller hand and the mainsail hand. This analysis was performed separately for each of the four variables: the X-axis, Y-axis, Z-axis, and vector magnitude. The analysis aimed to identify any significant differences in the complexity of hand motion across these axes.
6. To further investigate the significant effects observed in the one-way repeated measures ANOVA, a post hoc Tukey’s Honest Significant Difference test was performed. This allowed for targeted, pairwise comparisons between the conditions while upholding the appropriate statistical standards after the initial ANOVA analysis.

## 3. Results

To compute Approximate Entropy (ApEn) values from the motion time-series data, we experimented with various combinations of parameter values, specifically ‘m’ values ranging from 2 to 5, and ‘r’ values between 0.10 and 0.50. These computations were applied to the X-axis, Y-axis, Z-axis, and vector magnitude of both dominant and non-dominant hand motion data, resulting in a total of 24 parameter combinations for each ApEn analysis.

The mean and standard deviation (SD) of ApEn values for tiller (dominant) and mainsail (non-dominant) hand acceleration patterns are presented in Table A1. The analysis of variance (ANOVA) was conducted to assess the impact of both tiller and mainsail hands, along with factors M and R, on ApEn values across four variables: X, Y, Z, and vector magnitude (Vm). The results are detailed below.

For variable X, the ANOVA revealed no statistically significant difference between the tiller and mainsail hands (*p* = 0.906). This high *p*-value suggests that the mean ApEn values for variable X do not differ significantly between the two. However, the factor M exhibited a highly significant effect on X, with a *p*-value of less than 0.001 (*p* = 4.03 × 10^−5^), indicating that different levels of M contribute substantially to the variance in X.

While the analysis of variable Y yielded a *p*-value of 0.0851, suggesting a potential difference between tiller and mainsail hand acceleration patterns, this difference did not reach statistical significance at the conventional α = 0.05 level, indicating that any difference in mean ApEn values between both hands may be minor. In contrast, the factor M had a highly significant impact on Y (*p* = 7.41 × 10^−5^), similar to its effect on X.

Variable Z showed a statistically significant difference between the tiller and mainsail hands (*p* = 0.000673), highlighting a strong difference in ApEn values between the two hands. This finding suggests that the complexity of hand acceleration patterns, as measured by ApEn, varies significantly between the tiller and mainsail hands on the Z-axis. Additionally, the factor M had a highly significant effect on Z, with an extremely low *p*-value (*p* = 5.36 × 10^−7^), further emphasizing its influence. The small residuals observed indicate that the model explains most of the variance in Z.

For variable Vm, the ANOVA results indicated no statistically significant difference between the tiller and mainsail hands (*p* = 0.145). This suggests that the mean ApEn values for vector magnitude do not differ significantly between the two groups. Nevertheless, similar to the other variables, the factor M demonstrated a highly significant effect on Vm (*p* = 2.07 × 10^−5^), suggesting that M consistently influences the variance across different axes.

Given the significant ANOVA result for variable Z, a post hoc Tukey’s Honest Significant Difference (HSD) test was conducted. The Tukey’s HSD test confirmed the ANOVA results, revealing that the ApEn values for the mainsail (non-dominant) hand were significantly higher than those for the tiller (dominant) hand on the Z-axis (*p* = 0.000673). These findings suggest that the mainsail hand exhibits greater variability and less predictability in its acceleration patterns during the sailing simulation task, in contrast to the dominant tiller hand.

The influence of the ‘r’ parameter on ApEn values was examined across all measured variables. As expected, elevating the ‘r’ parameter, which widens the tolerance for resemblance between data sequences, typically led to lower Approximate Entropy values. This was observed consistently across all parameters such as the X-axis tiller hand mean (decreasing from 0.272 at R0.1 to 0.028 at R0.4) and Y-axis mainsail hand mean (decreasing from 0.282 at R0.1 to 0.020 at R0.4).

Table A1—ApEn values for the X-axis, Y-axis, Z-axis, and vector magnitude as a function of the 20-point input parameter combinations m and r, for dominant and non-dominant hands, in terms of the mean and standard deviation.

## 4. Discussion

This study explores entropy variations between tiller (dominant) and mainsail (non-dominant) hand acceleration patterns within a multi-directional sailing simulation task. Using ApEn, a well-established tool for assessing complexity and predictability in time-series data, our research reveals subtle yet significant differences in the regularity and complexity of acceleration profiles in this simulated environment.

Our study introduces a task with three-dimensional freedom, facilitating hand acceleration analyses across the Y-, X-, and Z-axes. This innovative sailing simulation necessitated a more thorough investigation of acceleration, particularly as the Z-axis exhibited notable differences in the complexity of acceleration between the tiller and mainsail hands. While the present sailing simulation task shared some commonalities with traditional reaching paradigms, it incorporated additional elements to mitigate variability and maintain consistency with established reaching protocols. Specifically, the simulation featured a “ghost” boat that provided a target course, encouraging participants to minimize deviations from the pre-defined path. This is similar to reaching tasks, where hand movements typically exhibit uniformity and follow pre-defined trajectories with minimal variability. In contrast, the dynamic nature of sailing, which requires continuous adjustments due to factors such as wind and boat behavior, does result in less predictable and more varied hand movements. As a result, previous research indicating parameters used in reaching tasks may not apply to the dynamic nature of sailing. Our research highlights the need for sailing-specific parameters to capture these nuanced hand accelerations, which differ based on sailing complexity.

Our analysis of the sailing task revealed distinct entropy variations, with notably higher ApEn values in the mainsail (non-dominant) hand. These elevated values indicate greater irregularity and unpredictability in the mainsail hand’s acceleration patterns, likely due to the complex demands of sailing. The tiller (dominant) hand, receiving more attention and training in daily activities, exhibited enhanced motor control and precision, reflected in its lower ApEn values across the X-, Y-, and Z-axes, as well as the vector magnitude. Whilst there are no previous studies on entropy for both hands in sailing, research has indicated that inexperienced sailors exhibit a limited ability to adapt to changing conditions, which may lead to less coordinated acceleration patterns between their hands during maneuvers [25]. This is particularly relevant as inexperienced sailors often struggle with the complexities of sail handling, resulting in more pronounced and exaggerated acceleration patterns of the mainsail hand to manage the sail effectively [26]. While expert sailors exhibit more refined and coordinated acceleration profiles, novices are likely to overcompensate with their mainsail hand, leading to a higher incidence of upper-limb overuse injuries [27]. Therefore, our results align with previous findings suggesting that inexperienced sailors primarily use their mainsail hand more than their tiller hand during sailing.

The parameter ‘r’ in the Approximate Entropy algorithm represents the tolerance or similarity threshold used to determine whether two data sequences are considered sufficiently alike to be classified as matching or resembling one another. The findings indicated that increasing the ‘r’ parameter typically resulted in a more smooth and less complex representation of the data. However, the rate of ApEn decreasing varied depending on the specific parameter and axis, indicating differential sensitivity to changes in ‘r’. For instance, while the X-axis tiller hand mean exhibited a substantial decrease, the Vm tiller hand mean showed a less pronounced reduction. These findings highlight the importance of considering parameter-specific responses to ‘r’ when interpreting ApEn values and underscore that larger similarity thresholds can mask subtle variations in data complexity. As the sailing task itself is very complex but also very sensitive, maintaining a low ‘r’ value is vital to ensure the identification of nuanced differences in hand acceleration patterns. However, if this task was less sensitive and required larger, slower acceleration profiles, a larger ‘r’ value may be more appropriate to smooth over the natural variability and identify the broader patterns.

The parameter ‘m’ in the Approximate Entropy algorithm represents the length of the sequences being compared to assess similarity within the data. The analysis revealed that increasing the ‘m’ parameter generally led to lower ApEn values, reflecting a more stringent and less complex representation of the data. These findings also emphasize the importance of carefully selecting the ‘m’ parameter to capture the appropriate level of detail in the data. For tasks that require sensitivity to subtle variations in acceleration patterns, such as the sailing simulation task described, a lower ‘m’ value for the Approximate Entropy algorithm may be necessary. This is because the sailing task involves irregular acceleration, necessitating the ability to capture subtle details in the data. Conversely, for other tasks where the goal is to identify broader, more consistent acceleration profiles, a higher ‘m’ value may be more appropriate, as it can provide a less complex, smoother representation of the data.

The Approximate Entropy metric has demonstrated its effectiveness in analyzing motion time-series data, as evidenced by its ability to distinguish between the tiller and mainsail hand acceleration patterns, particularly on the Z-axis, as confirmed by the post hoc Tukey’s HSD test [28]. Additionally, ApEn was capable of capturing a wide range of hand acceleration profiles, from subtle, precise changes to more broad, sweeping shifts. Experimenting with ‘m’ values (2, 3, 4, and 5) and ‘r’ values ranging from 0.10 to 0.50 provided insights. The factors ‘m’ and ‘r’ consistently had a significant impact across all variables (X, Y, Z, Vm), highlighting their influence on the complexity of hand acceleration patterns. These findings offer valuable guidance for researchers aiming to optimize ‘m’ and ‘r’ parameter selections when using ApEn to gauge hand acceleration complexity in sailing. Our research suggests that low ‘m’ (2) and ‘r’ (0.1) parameter values are sufficient to ensure an adequate number of sequence vectors within the tolerance for estimating conditional probabilities in our sailing simulation task.

The present study lays the foundation and key results for future research investigating the use of IMUs in analyzing motion time-series data. Such analyses could benefit from the application of Approximate Entropy due to its capacity to detect complexities and irregularities in multi-directional sailing tasks. The parameters selected for ApEn calculation should be carefully considered and varied to explore the sensitivity of the method in detecting differences in hand acceleration profiles. Our findings indicate that in the context of sailing using a VSail simulator, selecting and adjusting ApEn parameters diligently are essential. Optimal parameter combinations, such as m = 2, 3, 4, and 5 with r = 0.15, 0.20, 0.30, 0.40, and 0.50, were identified as effective in detecting nuances in hand acceleration differences and should be specifically utilized when examining reaching data within this domain. By introducing a multi-directional reaching task using sailing, our research offers valuable opportunities for functional diagnostics and assessments for multiple populations. Future studies could also explore more flexible hand-task assignments or how more experienced sailors adapt to sailing operations without hand assignments.

## 5. Conclusions

In conclusion, this study highlights the importance of Approximate Entropy (ApEn) as a tool for analyzing the complexities of hand acceleration patterns in a sailing simulation task. The findings reveal significant differences in the regularity and predictability of acceleration patterns between the tiller (dominant) and mainsail (non-dominant) hands, with the mainsail hand exhibiting higher ApEn values, suggestive of greater variability and complexity. The findings emphasize the importance of meticulously selecting and adjusting the ‘m’ and ‘r’ parameters in an Approximate Entropy analysis to effectively discern the subtleties of motor control during these types of tasks. The study establishes a foundation for future research, particularly in the context of using IMUs to explore motion time-series data in multi-directional tasks. By applying these insights, further exploration into hand acceleration dynamics can enhance our understanding of motor control in both healthy and clinical populations.

## Data Availability

The raw data supporting the conclusions of this article will be made available by the authors on request.

## Appendix A

**Table A1:** The mean and standard deviation of ApEn values for the X-axis, Y-axis, Z-axis, and vector magnitude of motion time-series data, across a range of ‘m’ and ‘r’ parameter combinations, for both tiller (dominant) and mainsail (non-dominant) hand movements.

M	R	Approximate Entropy															
		X				Y				Z				Vm			
		Dominant		Non-Dominant		Dominant		Non-Dominant		Dominant		Non-Dominant		Dominant		Non-Dominant	
		Mean	SD	Mean	SD	Mean	SD	Mean	SD	Mean	SD	Mean	SD	Mean	SD	Mean	SD
**2**	0.1	0.272	0.052	0.283	0.068	0.261	0.028	0.283	0.054	0.204	0.022	0.212	0.028	0.428	0.055	0.453	0.086
	0.15	0.143	0.038	0.147	0.043	0.136	0.029	0.148	0.038	0.095	0.014	0.103	0.023	0.220	0.043	0.233	0.056
	0.2	0.083	0.023	0.091	0.033	0.075	0.021	0.090	0.026	0.054	0.009	0.059	0.016	0.126	0.028	0.143	0.040
	0.3	0.049	0.014	0.055	0.024	0.042	0.014	0.052	0.017	0.029	0.008	0.035	0.014	0.071	0.018	0.086	0.027
	0.4	0.029	0.012	0.037	0.018	0.027	0.010	0.034	0.011	0.017	0.007	0.019	0.008	0.044	0.014	0.055	0.018
**3**	0.1	0.247	0.054	0.248	0.060	0.245	0.039	0.254	0.048	0.187	0.019	0.197	0.034	0.396	0.063	0.407	0.081
	0.15	0.130	0.036	0.136	0.041	0.121	0.027	0.129	0.037	0.083	0.012	0.092	0.022	0.198	0.041	0.209	0.057
	0.2	0.073	0.022	0.082	0.033	0.066	0.020	0.078	0.024	0.046	0.008	0.051	0.015	0.109	0.027	0.126	0.040
	0.3	0.040	0.013	0.050	0.025	0.036	0.013	0.046	0.015	0.025	0.007	0.030	0.012	0.060	0.018	0.076	0.027
	0.4	0.025	0.010	0.035	0.016	0.023	0.009	0.030	0.010	0.015	0.006	0.017	0.007	0.038	0.013	0.050	0.018
**4**	0.1	0.227	0.049	0.223	0.053	0.228	0.035	0.233	0.043	0.175	0.018	0.183	0.034	0.367	0.057	0.371	0.073
	0.15	0.113	0.034	0.117	0.040	0.108	0.024	0.113	0.034	0.079	0.019	0.083	0.022	0.177	0.039	0.184	0.053
	0.2	0.064	0.021	0.072	0.029	0.059	0.018	0.069	0.022	0.040	0.007	0.045	0.014	0.097	0.025	0.111	0.036
	0.3	0.035	0.011	0.046	0.021	0.032	0.012	0.041	0.014	0.022	0.006	0.025	0.009	0.053	0.016	0.067	0.025
	0.4	0.021	0.009	0.032	0.015	0.020	0.008	0.027	0.010	0.014	0.006	0.015	0.006	0.033	0.012	0.045	0.017
**5**	0.1	0.209	0.046	0.199	0.053	0.209	0.038	0.207	0.052	0.160	0.029	0.165	0.042	0.337	0.062	0.332	0.082
	0.15	0.100	0.029	0.107	0.039	0.098	0.022	0.108	0.031	0.069	0.010	0.077	0.021	0.157	0.033	0.171	0.051
	0.2	0.058	0.019	0.063	0.027	0.054	0.016	0.062	0.021	0.037	0.006	0.041	0.013	0.088	0.023	0.099	0.033
	0.3	0.033	0.011	0.042	0.018	0.030	0.012	0.038	0.011	0.020	0.006	0.024	0.008	0.050	0.016	0.063	0.021
	0.4	0.019	0.008	0.029	0.014	0.018	0.008	0.025	0.009	0.012	0.005	0.014	0.006	0.029	0.011	0.041	0.015
